# [μ-3-(Methyl­sulfan­yl)benzene-1,2-di­thiol­ato-1:2κ^4^ *S*,*S*′:*S*,*S*′]bis­[tri­carbonyl­iron(I)]

**DOI:** 10.1107/S1600536813009860

**Published:** 2013-04-13

**Authors:** Yong Yang, Ning Wang, Lin Chen

**Affiliations:** a State Key Laboratory of Fine Chemicals, Dalian University of Technology (DUT), Dalian 116024, People’s Republic of China; b School of Chemistry and Chemical Engineering, Henan University of Technology, Zhengzhou 450001, People’s Republic of China

## Abstract

The title compound, [Fe_2_(C_7_H_6_S_3_)(CO)_6_], was prepared as a biomimic for the active site of [FeFe]-hydrogenases. The central Fe_2_S_2_ core is in a butterfly conformation and each Fe^I^ atom has a pseudo-square-pyramidal coordination by three O atoms and two S atoms. The Fe—Fe distance is 2.471 (2) Å and the dihedral angle between the two Fe—S—Fe planes is 78.96 (7)°. The least-squares plane through the –S(C_7_H_6_S)S– bridge nearly bis­ects the mol­ecular structure: except for the two Fe(CO)_3_ units, all atoms are in this plane with an average deviation from the plane of 0.028 (3) Å. In the crystal, the mol­ecules are linked into chains along [001] by C—H⋯π(arene) inter­actions.

## Related literature

For general background to [FeFe]-hydrogenases, see: Capon *et al.* (2009 ▶); Tard & Pickett (2009 ▶). For the crystal structure of the natural enzyme, see: Peters *et al.* (1998 ▶); Nicolet *et al.* (1999 ▶). For related structures and the synthesis, see: Maiolo *et al.* (1981 ▶); Wang *et al.* (2005 ▶); Dong *et al.* (2006 ▶).
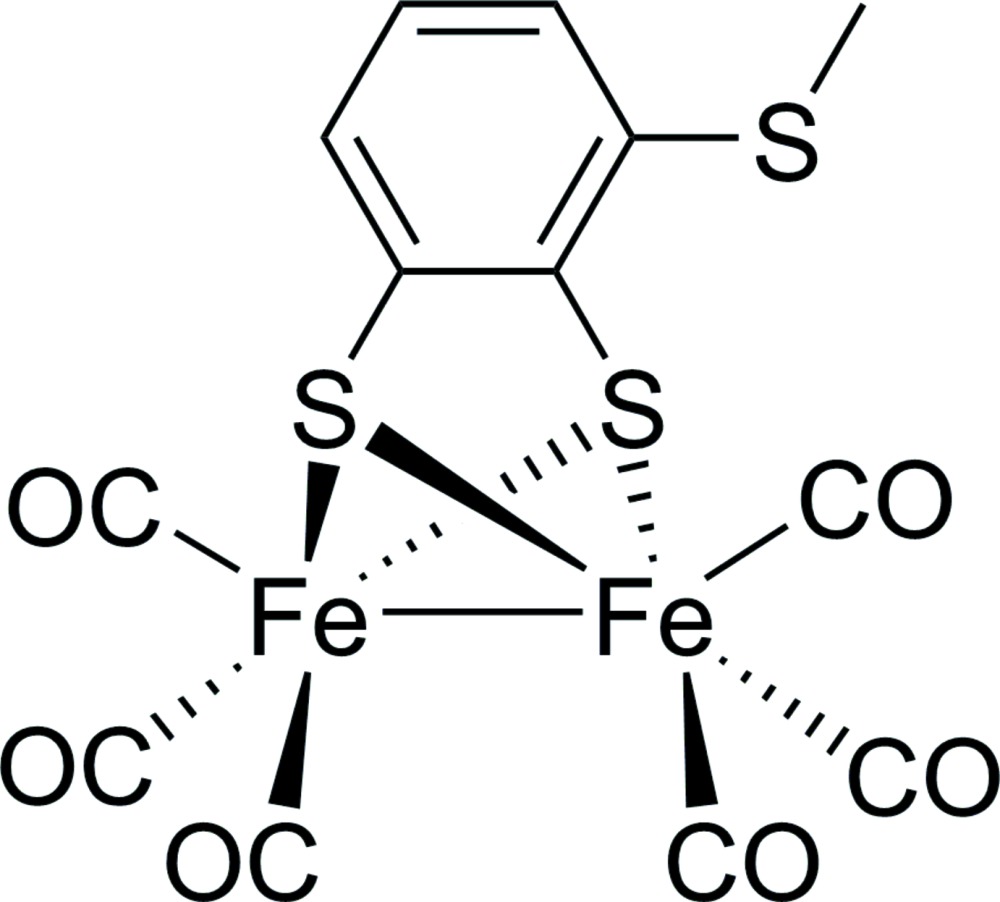


## Experimental

### 

#### Crystal data

[Fe_2_(C_7_H_6_S_3_)(CO)_6_]
*M* *_r_* = 466.06
Monoclinic, 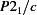

*a* = 16.531 (17) Å
*b* = 7.975 (8) Å
*c* = 13.047 (13) Å
β = 92.055 (13)°
*V* = 1719 (3) Å^3^
*Z* = 4
Mo *K*α radiation
μ = 2.08 mm^−1^
*T* = 295 K
0.39 × 0.24 × 0.08 mm

#### Data collection

Bruker APEXII CCD diffractometer
Absorption correction: multi-scan (*SADABS*; Bruker, 2001 ▶) *T*_min_ = 0.555, *T*_max_ = 0.847
8260 measured reflections
3024 independent reflections
2098 reflections with *I* > 2σ(*I*)
*R*_int_ = 0.055

#### Refinement

*R*[*F* ^2^ > 2σ(*F* ^2^)] = 0.036
*wR*(*F* ^2^) = 0.084
*S* = 0.97
3024 reflections
217 parameters
H-atom parameters constrained
Δρ_max_ = 0.44 e Å^−3^
Δρ_min_ = −0.31 e Å^−3^

### 

Data collection: *APEX2* (Bruker, 2007 ▶); cell refinement: *SAINT-Plus* (Bruker, 2007 ▶); data reduction: *SAINT-Plus*; program(s) used to solve structure: *SHELXS97* (Sheldrick, 2008 ▶); program(s) used to refine structure: *SHELXL97* (Sheldrick, 2008 ▶); molecular graphics: *SHELXTL* (Sheldrick, 2008 ▶); software used to prepare material for publication: *SHELXL97*.

## Supplementary Material

Crystal structure: contains datablock(s) I, global. DOI: 10.1107/S1600536813009860/vn2067sup1.cif

Structure factors: contains datablock(s) I. DOI: 10.1107/S1600536813009860/vn2067Isup2.hkl

Additional supplementary materials:  crystallographic information; 3D view; checkCIF report

## Figures and Tables

**Table 1:** Selected bond lengths (Å)

Fe1—C1	1.789 (5)
Fe1—C2	1.786 (4)
Fe1—C3	1.788 (5)
Fe1—S1	2.2695 (19)
Fe1—S2	2.253 (2)
Fe2—C4	1.789 (5)
Fe2—C5	1.781 (4)
Fe2—C6	1.784 (5)
Fe2—S1	2.2763 (19)
Fe2—S2	2.263 (2)

**Table 2:** C—H⋯π(arene) inter­action geometry (Å, °) *Cg*1 is the centroid of the C7–C12 ring.

*D*—H⋯*A*	*D*—H	H⋯*A*	*D*⋯*A*	*D*—H⋯*A*
C13—H13*A*⋯*Cg*1^i^	0.96	2.69	3.590 (5)	157

Symmetry code: (i) 
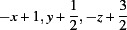
.

**Fig. 1.**
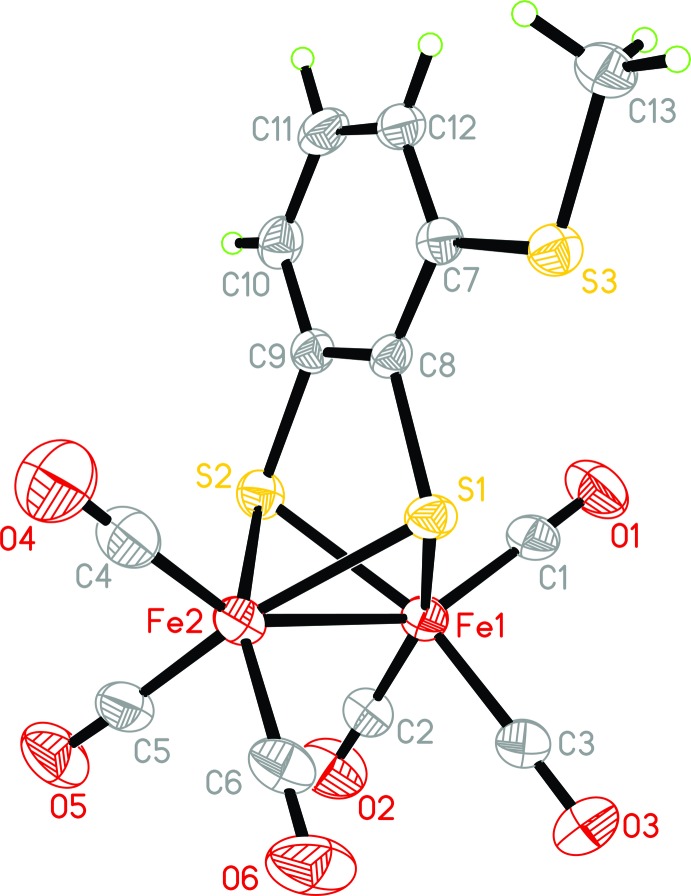
Molecular structure of the title compound, with displacement ellipsoids drawn at the 30% probability level.

## supplementary crystallographic information

### Comment

[FeFe]-hydrogenases are important enzymes in numerous microorganisms, which can catalyse hydrogen evolution or uptake. Crystallographic and IR spectroscopic studies on the two types of [FeFe]Hases, CpI (*Clostridium pasteurianum*) and DdH (*Desulfovibrio desulfuricans*), revealed that the active site of [FeFe]-hydrogenases is comprised of a 2Fe2S butterfly structure, which contains diatomic ligands CO and CN^-^, a cysteinyl-S ligand connecting to a 4Fe4S subcluster, and a three-atom linker (–CH_2_XCH_2_–, *X* = CH_2_,NH or O) bridged between the two S atoms of the Fe_2_S_2_ H-cluster (Capon *et al.*, 2009, Tard & Pickett, 2009). It was found that the introduction of a rigid and conjugate bridge to the Fe_2_S_2_ complexes could make the electrochemical properties of the complexes apparently different from the Fe_2_S_2_ complexes with flexible bridges (Capon *et al.*, 2009).

The structure of the title compound resembles the active site of [FeFe]-hydrogenases, with a butterfly architectonic 2Fe2S core and the usual distorted square-pyramidal geometry around the iron center (Wang *et al.*, 2005, Dong *et al.*, 2006). The length of the Fe–Fe bond [2.471 (2) Å] is slightly shorter than those in the structures of natural enzymes (*ca* 2.6 Å) (Peters *et al.*, 1998, Nicolet *et al.*, 1999). The dihedral angle between two Fe–S–Fe planes is 78.96 (7) °. The rigid dithiolate bridge is a special feature for the title compound. The molecular structure has an internal pseudo-mirror plane passing through the aromatic ring. The calculated plane of the SRS bridge is nearly a bisecting plane of the molecular structure. Except for two Fe(CO)_3_ units all atoms are in the plane with the average deviation of 0.0280 Å. The deviations of the iron atoms from the SRS basal plane are 1.266 (5) Å for Fe(1) and 1.205 (5) Å for Fe(2). The molecular structure of the title compound is shown in Figure 1.

### Experimental

All reactions and operations related to the title compound were carried out under a dry, prepurified nitrogen atmosphere with standard Schlenk techniques. All solvents were dried and distilled prior to use according to standard methods. The starting material 3-(methylthio)benzene-1,2-dilthiol was prepared by a similar procedure according to the literature (Maiolo *et al.*, 1981). 3-(Methylthio)benzene-1,2-dilthiol (1 mmol, 0.188 g) and freshly synthesized Fe_3_(CO)_12_ (1 mmol, 0.503 g) were refluxed in toluene under N_2_ atmosphere for 3 h. The color of the solution changed gradually from dark green to dark red. The solvent was removed under reduced pressure. The residue was purified by column chromatography on silica gel using hexane as eluent to give the title compound as a red solid (0.294 g, 63%). A single-crystal suitable for X-ray study was obtained by slow evaporation of CH_2_Cl_2_/hexane (1:10, *v/v*) solution at room temperature. IR (CH_2_Cl_2_, cm^-1^): *ν*(CO) 2075 (*m*), 2045 (*s*), 1999 (*versus*); ^1^H NMR (400 MHz, CDCl_3_): *δ* 6.92 (s, 1H, C_6_H_3_), 6.60 (s, 1H, C_6_H_3_), 6.53 (s, 1H, C_6_H_3_), 2.41 (s, 3H, SCH_3_).

### Refinement

Hydrogen atoms were positioned geometrically and refined as riding atoms, with C–H = 0.93 (CH) and 0.96 (CH_3_) Å and with *U*_iso_(H) = 1.2 (1.5 for methyl) *U*_eq_(C).

### Crystal data

**Table:**

[Fe_2_(C_7_H_6_S_3_)(CO)_6_]	*F*(000) = 928
*M**_r_* = 466.06	*D*_x_ = 1.801 Mg m^−^^3^
Monoclinic, *P*2_1_/*c*	Mo *K*α radiation, λ = 0.71073 Å
Hall symbol: -P 2ybc	Cell parameters from 2629 reflections
*a* = 16.531 (17) Å	θ = 2.5–27.1°
*b* = 7.975 (8) Å	µ = 2.08 mm^−^^1^
*c* = 13.047 (13) Å	*T* = 295 K
β = 92.055 (13)°	Block, red
*V* = 1719 (3) Å^3^	0.39 × 0.24 × 0.08 mm
*Z* = 4	

### Data collection

**Table:**

Bruker APEXII CCD diffractometer	3024 independent reflections
Radiation source: fine-focus sealed tube	2098 reflections with *I* > 2σ(*I*)
Graphite monochromator	*R*_int_ = 0.055
φ and ω scans	θ_max_ = 25.0°, θ_min_ = 2.5°
Absorption correction: multi-scan (*SADABS*; Bruker, 2001)	*h* = −19→18
*T*_min_ = 0.555, *T*_max_ = 0.847	*k* = −9→9
8260 measured reflections	*l* = −15→13

### Refinement

**Table:**

Refinement on *F*^2^	Primary atom site location: structure-invariant direct methods
Least-squares matrix: full	Secondary atom site location: difference Fourier map
*R*[*F*^2^ > 2σ(*F*^2^)] = 0.036	Hydrogen site location: inferred from neighbouring sites
*wR*(*F*^2^) = 0.084	H-atom parameters constrained
*S* = 0.97	*w* = 1/[σ^2^(*F*_o_^2^) + (0.0341*P*)^2^] where *P* = (*F*_o_^2^ + 2*F*_c_^2^)/3
3024 reflections	(Δ/σ)_max_ < 0.001
217 parameters	Δρ_max_ = 0.44 e Å^−^^3^
0 restraints	Δρ_min_ = −0.31 e Å^−^^3^

### Special details

**Table:**

Geometry. All e.s.d.'s (except the e.s.d. in the dihedral angle between two l.s. planes) are estimated using the full covariance matrix. The cell e.s.d.'s are taken into account individually in the estimation of e.s.d.'s in distances, angles and torsion angles; correlations between e.s.d.'s in cell parameters are only used when they are defined by crystal symmetry. An approximate (isotropic) treatment of cell e.s.d.'s is used for estimating e.s.d.'s involving l.s. planes.
Refinement. Refinement of *F*^2^ against ALL reflections. The weighted *R*-factor *wR* and goodness of fit *S* are based on *F*^2^, conventional *R*-factors *R* are based on *F*, with *F* set to zero for negative *F*^2^. The threshold expression of *F*^2^ > σ(*F*^2^) is used only for calculating *R*-factors(gt) *etc*. and is not relevant to the choice of reflections for refinement. *R*-factors based on *F*^2^ are statistically about twice as large as those based on *F*, and *R*- factors based on ALL data will be even larger.

### Fractional atomic coordinates and isotropic or equivalent isotropic displacement parameters (Å^2^)

**Table:**

	*x*	*y*	*z*	*U*_iso_*/*U*_eq_	
Fe1	0.17590 (2)	0.29795 (7)	0.64150 (4)	0.04642 (17)	
Fe2	0.18906 (3)	0.54555 (7)	0.52941 (4)	0.04814 (18)	
S3	0.46296 (5)	0.53167 (12)	0.73543 (7)	0.0462 (2)	
S2	0.22860 (5)	0.28468 (12)	0.48479 (7)	0.0475 (2)	
S1	0.27674 (5)	0.48911 (12)	0.66349 (7)	0.0446 (2)	
C8	0.35707 (16)	0.3899 (4)	0.5983 (2)	0.0354 (7)	
C10	0.3893 (2)	0.2051 (4)	0.4606 (3)	0.0481 (9)	
H10A	0.3730	0.1375	0.4055	0.058*	
C2	0.0831 (2)	0.2118 (5)	0.5909 (3)	0.0560 (10)	
C9	0.33421 (18)	0.2915 (4)	0.5159 (3)	0.0401 (8)	
C5	0.1075 (2)	0.5260 (5)	0.4373 (4)	0.0605 (11)	
C1	0.21760 (19)	0.1120 (6)	0.6991 (3)	0.0564 (10)	
C7	0.43793 (17)	0.4062 (4)	0.6280 (3)	0.0377 (8)	
C11	0.4698 (2)	0.2221 (5)	0.4897 (3)	0.0514 (9)	
H11A	0.5086	0.1648	0.4535	0.062*	
C12	0.49397 (19)	0.3216 (4)	0.5709 (3)	0.0449 (9)	
H12A	0.5488	0.3324	0.5879	0.054*	
O2	0.02428 (15)	0.1593 (4)	0.5570 (3)	0.0859 (10)	
O1	0.24308 (17)	−0.0076 (4)	0.7338 (3)	0.0867 (11)	
C4	0.2506 (2)	0.6882 (6)	0.4606 (4)	0.0700 (12)	
O5	0.05695 (18)	0.5075 (4)	0.3771 (3)	0.0918 (11)	
O6	0.09707 (19)	0.7802 (4)	0.6518 (3)	0.0961 (12)	
O4	0.2905 (2)	0.7821 (5)	0.4201 (3)	0.1088 (13)	
O3	0.09360 (19)	0.4359 (5)	0.8164 (3)	0.0994 (12)	
C6	0.1338 (2)	0.6903 (5)	0.6042 (4)	0.0643 (12)	
C3	0.1254 (2)	0.3805 (6)	0.7494 (4)	0.0631 (11)	
C13	0.57037 (19)	0.5072 (4)	0.7506 (3)	0.0543 (10)	
H13A	0.5900	0.5721	0.8082	0.082*	
H13B	0.5956	0.5454	0.6897	0.082*	
H13C	0.5831	0.3911	0.7619	0.082*	

### Atomic displacement parameters (Å^2^)

**Table:**

	*U*^11^	*U*^22^	*U*^33^	*U*^12^	*U*^13^	*U*^23^
Fe1	0.0379 (3)	0.0561 (4)	0.0452 (3)	−0.0011 (2)	0.0000 (2)	0.0002 (3)
Fe2	0.0429 (3)	0.0491 (4)	0.0522 (4)	0.0050 (2)	−0.0029 (2)	−0.0011 (3)
S3	0.0421 (4)	0.0487 (6)	0.0479 (6)	−0.0040 (4)	0.0011 (4)	−0.0067 (5)
S2	0.0480 (4)	0.0519 (6)	0.0422 (6)	−0.0004 (4)	−0.0046 (4)	−0.0077 (5)
S1	0.0379 (4)	0.0538 (6)	0.0423 (6)	0.0033 (4)	0.0016 (4)	−0.0123 (4)
C8	0.0392 (15)	0.036 (2)	0.0313 (19)	0.0026 (15)	0.0060 (13)	−0.0023 (16)
C10	0.061 (2)	0.046 (2)	0.038 (2)	0.0035 (18)	0.0044 (17)	−0.0051 (18)
C2	0.052 (2)	0.058 (3)	0.057 (3)	0.0055 (19)	−0.0012 (18)	0.002 (2)
C9	0.0433 (16)	0.040 (2)	0.037 (2)	0.0048 (15)	0.0035 (14)	0.0003 (17)
C5	0.052 (2)	0.061 (3)	0.068 (3)	0.0124 (19)	−0.001 (2)	−0.002 (2)
C1	0.0372 (17)	0.070 (3)	0.061 (3)	−0.0023 (19)	−0.0012 (17)	−0.001 (2)
C7	0.0405 (16)	0.0300 (19)	0.043 (2)	−0.0003 (14)	0.0051 (14)	0.0067 (16)
C11	0.056 (2)	0.050 (2)	0.050 (3)	0.0106 (18)	0.0194 (17)	0.003 (2)
C12	0.0416 (17)	0.046 (2)	0.048 (2)	0.0045 (16)	0.0079 (15)	0.0058 (19)
O2	0.0549 (15)	0.087 (2)	0.115 (3)	−0.0205 (15)	−0.0156 (17)	−0.004 (2)
O1	0.0665 (18)	0.085 (2)	0.108 (3)	0.0094 (17)	−0.0127 (18)	0.028 (2)
C4	0.062 (2)	0.069 (3)	0.079 (3)	0.006 (2)	−0.005 (2)	0.006 (3)
O5	0.0720 (19)	0.109 (3)	0.092 (3)	0.0123 (17)	−0.034 (2)	−0.008 (2)
O6	0.083 (2)	0.088 (3)	0.117 (3)	0.0311 (18)	−0.0015 (19)	−0.043 (2)
O4	0.101 (2)	0.096 (3)	0.130 (4)	−0.025 (2)	0.021 (2)	0.030 (3)
O3	0.087 (2)	0.145 (3)	0.068 (3)	0.028 (2)	0.0250 (19)	−0.007 (2)
C6	0.048 (2)	0.064 (3)	0.080 (3)	0.004 (2)	−0.011 (2)	−0.005 (3)
C3	0.0422 (19)	0.087 (3)	0.060 (3)	0.005 (2)	0.0038 (19)	0.007 (3)
C13	0.0432 (18)	0.055 (3)	0.064 (3)	−0.0049 (16)	−0.0044 (17)	−0.001 (2)

### Geometric parameters (Å, º)

**Table:**

Fe1—C1	1.789 (5)	C8—C9	1.373 (4)
Fe1—C2	1.786 (4)	C10—C9	1.368 (5)
Fe1—C3	1.788 (5)	C10—C11	1.378 (5)
Fe1—Fe2	2.471 (2)	C10—H10A	0.9300
Fe1—S1	2.2695 (19)	C11—C12	1.372 (5)
Fe1—S2	2.253 (2)	C11—H11A	0.9300
Fe2—C4	1.789 (5)	C12—H12A	0.9300
Fe2—C5	1.781 (4)	C13—H13A	0.9600
Fe2—C6	1.784 (5)	C13—H13B	0.9600
Fe2—S1	2.2763 (19)	C13—H13C	0.9600
Fe2—S2	2.263 (2)	O3—C3	1.127 (5)
C1—O1	1.131 (4)	O6—C6	1.139 (5)
C2—O2	1.133 (4)	S1—C8	1.787 (3)
C4—O4	1.140 (5)	S2—C9	1.779 (3)
C5—O5	1.135 (5)	S3—C7	1.759 (4)
C7—C12	1.385 (5)	S3—C13	1.790 (4)
C8—C7	1.385 (4)		
			
C2—Fe1—C3	90.77 (19)	C8—S1—Fe1	101.39 (13)
C2—Fe1—C1	98.59 (17)	C8—S1—Fe2	100.67 (13)
C3—Fe1—C1	99.1 (2)	Fe1—S1—Fe2	65.86 (5)
C2—Fe1—S2	90.09 (15)	C9—C8—C7	120.6 (3)
C3—Fe1—S2	159.76 (15)	C9—C8—S1	115.9 (2)
C1—Fe1—S2	100.74 (14)	C7—C8—S1	123.4 (2)
C2—Fe1—S1	157.30 (13)	C9—C10—C11	117.3 (3)
C3—Fe1—S1	90.84 (14)	C9—C10—H10A	121.3
C1—Fe1—S1	103.50 (13)	C11—C10—H10A	121.3
S2—Fe1—S1	80.80 (5)	O2—C2—Fe1	178.5 (4)
C2—Fe1—Fe2	100.45 (13)	C10—C9—C8	122.1 (3)
C3—Fe1—Fe2	103.00 (16)	C10—C9—S2	122.0 (3)
C1—Fe1—Fe2	150.44 (12)	C8—C9—S2	115.9 (2)
S2—Fe1—Fe2	57.01 (5)	O5—C5—Fe2	177.1 (4)
S1—Fe1—Fe2	57.20 (5)	O1—C1—Fe1	178.4 (4)
C5—Fe2—C6	92.06 (19)	C8—C7—C12	117.4 (3)
C5—Fe2—C4	98.5 (2)	C8—C7—S3	118.3 (2)
C6—Fe2—C4	100.1 (2)	C12—C7—S3	124.3 (2)
C5—Fe2—S2	87.98 (13)	C12—C11—C10	121.5 (3)
C6—Fe2—S2	153.34 (15)	C12—C11—H11A	119.3
C4—Fe2—S2	106.31 (16)	C10—C11—H11A	119.3
C5—Fe2—S1	161.32 (14)	C11—C12—C7	121.0 (3)
C6—Fe2—S1	91.81 (15)	C11—C12—H12A	119.5
C4—Fe2—S1	98.82 (15)	C7—C12—H12A	119.5
S2—Fe2—S1	80.45 (5)	O4—C4—Fe2	177.5 (5)
C5—Fe2—Fe1	104.41 (14)	O6—C6—Fe2	178.4 (4)
C6—Fe2—Fe1	97.78 (16)	O3—C3—Fe1	178.5 (5)
C4—Fe2—Fe1	150.30 (13)	S3—C13—H13A	109.5
S2—Fe2—Fe1	56.64 (3)	S3—C13—H13B	109.5
S1—Fe2—Fe1	56.94 (5)	H13A—C13—H13B	109.5
C7—S3—C13	103.30 (16)	S3—C13—H13C	109.5
C9—S2—Fe1	101.52 (13)	H13A—C13—H13C	109.5
C9—S2—Fe2	101.73 (12)	H13B—C13—H13C	109.5
Fe1—S2—Fe2	66.35 (4)		

### Hydrogen-bond geometry (Å, º)

Cg1 is the centroid of the C7–C12 ring.

**Table:**

*D*—H···*A*	*D*—H	H···*A*	*D*···*A*	*D*—H···*A*
C13—H13*A*···*Cg*1^i^	0.96	2.69	3.590 (5)	157

Symmetry code: (i) −*x*+1, *y*+1/2, −*z*+3/2.
